# In Vitro Biological Properties Assessment of 3D-Printed Hydroxyapatite–Polylactic Acid Scaffolds Intended for Bone Regeneration

**DOI:** 10.3390/jfb16060218

**Published:** 2025-06-12

**Authors:** Eddy Shan, Cristina Chamorro, Ana Ferrández-Montero, Rosa M. Martin-Rodriguez, Begoña Ferrari, Antonio Javier Sanchez-Herencia, Leire Virto, María José Marín, Elena Figuero, Mariano Sanz

**Affiliations:** 1 Section of Periodontology, Faculty of Odontology, Complutense University of Madrid, 28040 Madrid, Spain; 2 Etiology and Therapy of Periodontal and Peri-implant Diseases (ETEP) Research Group, Complutense University of Madrid, 28040 Madrid, Spainmjmarin@ucm.es (M.J.M.); 3 Institute of Ceramics and Glass (ICV), Spanish National Research Council (CSIC), 28049 Madrid, Spainbferrari@icv.csic.es (B.F.);

**Keywords:** tissue engineering, hydroxyapatite-polylactic acid, in vitro, scaffold, additive manufacturing, bone regeneration

## Abstract

This study evaluated the biological performance in vitro of two 3D-printed hydroxyapatite (HA) and polylactic acid (PLA) composite scaffolds with two different infill densities (50% [HA-PLA50] and 70% [HA-PLA70]). Comparative analysis using MG-63 cell cultures evaluated the following: (1) integrity after exposure to various sterilization methods; (2) cell viability; (3) morphological characteristics; (4) cell proliferation; (5) cytotoxicity; (6) gene expression; and (7) protein synthesis. Ultraviolet radiation was the preferred sterilization method. Both scaffolds maintained adequate cell viability and proliferation over 7 days without significant differences in cytotoxicity. Notably, HA-PLA50 scaffolds demonstrated superior osteogenic potential, showing a significantly higher expression of collagen type I (COL1A1) and an increased synthesis of interleukins 6 and 8 (IL-6, IL-8) compared to HA-PLA70 scaffolds. While both scaffold types supported robust cell growth, the HA-PLA50 formulation exhibited enhanced bioactivity, suggesting a potential advantage for bone tissue engineering applications. These findings provide important insights for optimizing 3D-printed bone graft substitutes.

## 1. Introduction

Dimensional changes in the alveolar ridge after tooth loss can lead to significant bone remodeling during the first year of healing, often preventing the placement of dental implants in a prosthetically driven position [1]. These clinical situations are currently managed with bone augmentation interventions, either simultaneous or staged with implant placement. These interventions follow tissue engineering principles, utilizing biomaterials and signaling molecules and/or cells while adhering to wound healing bases, such as primary wound closure, angiogenesis, space maintenance, and stability of the blood clot [2].

The most common bone augmentation approach has been guided bone regeneration, based on the use of barrier membranes combined with bone grafts or bone replacement grafts (autologous, xenogeneic, or allogeneic grafts) to achieve bone regeneration by promoting cell homing while maintaining the needed space for bone augmentation [3,4]. However, when using autografts, either blocks or particulate, bone availability is limited by anatomical constraints, and its harvesting is usually associated with increased morbidity and faster resorption [5]. Similarly, conventional xenografts and allogeneic grafts usually present difficulties in clinical handling and poorer bioactivity and dimensional stability [6]. These limitations, together with the current trends in avoiding human or animal-derived biomaterials, have prompted the research community to develop synthetically derived bone replacement grafts, which would provide the adequate handling and biological and physic-mechanical properties for the bone regeneration of the alveolar ridges [7].

Different metals, polymers, ceramics, and composite materials have been evaluated as biomaterials for tissue engineering applications aimed at bone regeneration [8,9]. Among these, polymers such as polylactic acid (PLA) have been evaluated, since they are biocompatible, biodegradable, and widely used as biomaterials in medical implant devices, as they are medical grade biomaterials (approved by the US Food and Drug Administration for medical use) [10]. However, PLA is limited by low bioactivity [7,11], accelerated resorption rates, and the release of acidic concentrations during its degradation [12]. Combining PLA with bioceramics like hydroxyapatite (HA) enhances its osteogenic bioactivity and counteracts the acidic environment produced during the in vitro degradation of PLA [13,14].

In recent years, the use of natural biomaterials as particulate or block forms, is being replaced by tissue engineering approaches using synthetic biomaterials where the scaffolds mimic the extracellular matrix of native bone, providing the framework for cell proliferation, cell differentiation and vascularization, thus promoting bone regeneration [15]. Scaffold properties, such as porosity, elasticity, and stability, have been shown to influence cell differentiation [11,16]. In fact, increased porosity has been associated with increased vascularity and a better supply of oxygen and nutrients, manifested by increased alkaline phosphatase activity, calcium mineralization, and faster bone formation [17].

These new biomaterials-based scaffolds and their fabrication methods are also being investigated to determine their mechanical properties [7,18] in search of customized solutions in bone regeneration. With new developments in imaging techniques and 3D printing, computer-based, additively manufactured scaffolds have been designed and fabricated using electrospinning and fused filament fabrication techniques [19,20,21,22].

Thus, in this research, we aim to characterize the in vitro biological properties of customizable HA-PLA scaffolds intended for bone regeneration fabricated with two different infill densities (50% vs. 70%).

## 2. Materials and Methods

### 2.1. Study Design

This in vitro investigation employs seven assays to examine the biological properties of HA-PLA scaffolds at two infill densities (50% and 70%). Experimental MG-63 cell lines from human osteosarcoma are positive controls, while the cells growing on the well surface act as negative controls. Ethics approval was not required for this study as it did not involve human or animal subjects.

For each assay, three to six biological replicates were carried out (except for the sterilization method assay, which used two replicates). All scaffolds were seeded simultaneously at the initial time point, and at each study interval (24 h, 48 h, 72 h, and 7 days), they were assessed and discarded.

### 2.2. Fabrication and Design of Scaffolds

**Figure 1:**
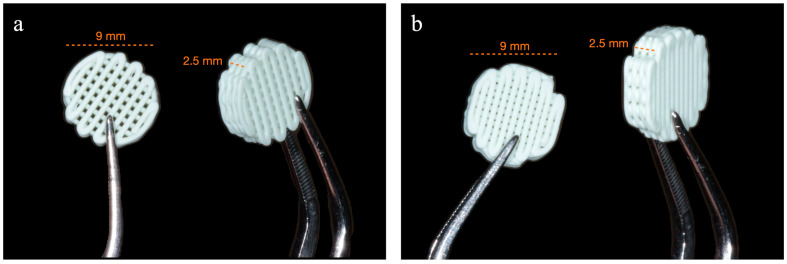
Scaffold designs, both with an analogous cylindrical geometry (9 mm diameter × 2.5 mm height). (**a**) HA-PLA50 scaffold design with a lower infill density (50%), exhibiting higher porosity due to wider spacing between deposited filaments. (**b**) HA-PLA70 scaffold design with a higher infill density (70%), which translates into a lower porosity and a denser internal architecture.

Commercial HA-containing filaments (FOss HA, COLFEED4Print S.L., Madrid, Spain) with a diameter of 1.75 mm and nominal particle content of 20 vol.% HA were used to print customizable 3D scaffolds. The filaments consist of highly dispersed HA particles within a thermoplastic matrix of PLA, fabricated using a colloidal method [23]. Cylindrical scaffolds with a diameter of 9 mm and a height of 2.5 mm were printed using the FFF (Fused Filament Fabrication) technique on an NX PRO Dual printer (Tumaker, Gipuzkoa, Spain) with a nozzle diameter of 0.4 mm. The cylinders were designed with a 3D freeware (Tinkercad, Autodesk^®^, San Francisco, CA, USA), while slicing was performed with Ultimaker Cura v5.3.1 software (Ultimaker, Utrecht, The Netherlands), selecting linear infill patterns. The printing process involved setting the bed and nozzle temperatures to 40 and 165 °C, respectively, with a filament feed rate of 20 mm/s. A standardized scaffold morphology was employed for both the 50% and 70% infill scaffolds, labeled HA-PLA50 and HA-PLA70 (Figure 1). The different infill densities tested are translated into differences in porosity, total material volume, and printing times. Specifically, a higher infill density reduces scaffold porosity and increases structural integrity and mechanical strength, albeit with longer printing times and greater material consumption, while low infill density results in higher porosity, which may enhance nutrient diffusion and cell infiltration at the expense of mechanical properties.

**Figure A1:**
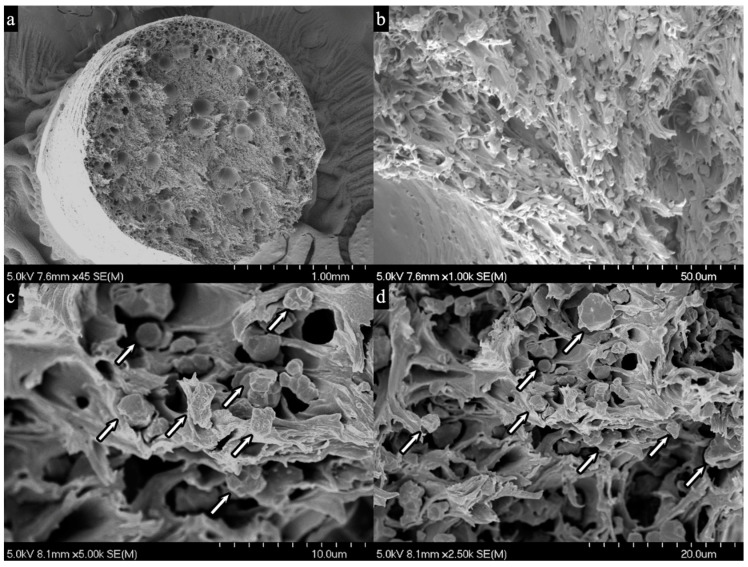
Surface of the fractured cross-section of the filament with the presence of closed pores of about 100 µm: (**a**) the filament is composed by the thermoplastic matrix, which is stretched due to strain during fracture; (**b**) individual particles of hydroxyapatite attached to the stretched walls of the polymer; (**c**,**d**) the arrows indicate inorganic particles highly dispersed into the PLA matrix without the presence of agglomerates as a consequence of the colloidal manufacturing approach.

Particle size ranges between 1 and 2 µm (Figure A1). The high dispersion of HA and the absence of agglomerates are expected to facilitate the osteoconductive effect until the complete biodegradation of the scaffold [24].

### 2.3. Cell Culture

Experimental MG-63 cell lines derived from human osteosarcoma (ATCC, CRL-1427) were utilized to evaluate the biological properties of the scaffolds. The cells were cultured in 75 cm^2^ flasks using Eagle’s Minimum Essential Medium (MEM) with Earle’s Balanced Salt Solution (Sigma-Aldrich, Saint Louis, MO, USA), supplemented with 10% fetal bovine serum (FBS) (Biowest, Nuaillé, France) and 1% penicillin–streptomycin (Gibco, Grand Island, NY, USA). The standard conditions for MG-63 cell incubation were set at 37 °C in a humidified atmosphere containing 5% CO_2_. Subcultures were performed once 80% cell confluence was reached. Cryopreservation of the cells was conducted using liquid nitrogen (−196 °C).

### 2.4. Experimental Assays

#### 2.4.1. Sterilization Method

Three methods of sterilization were compared: ultraviolet (UV) radiation (30 min per side), 70% ethanol (1 min + 3 PBS rinses) [25], and autoclave (121 °C, 1.10 atm, 20 min). For each method, two biological replicates of PLA-HA70 scaffolds were assessed in terms of scaffold integrity and success in attaining sterilization. Non-sterile scaffolds were used as a positive control, and culture plates with only media were used as negative controls.

The success of sterilization was evaluated though microbial culture. First, aliquots of each scaffold were obtained by submerging the scaffolds in 1 mL of PBS and vortexing for 1 min. Following this, a 1:10 (D1) dilution in PBS was prepared, and 100 µL of both the undiluted (D0) and D1 aliquots were cultured under aerobic conditions for 48 h and anaerobic conditions (80% N_2_, 10% H_2_, 10% CO_2_ at 37 °C for 72 h) using Blood Agar Base No. 2 with 5% horse blood and Hemine/menadione culture media (Oxoid no. 2, Oxoid, Basingstoke, UK). The presence or absence of bacterial or fungal growth was determined by visual inspection under magnifying loupes. The integrity of the scaffolds after the sterilization process was evaluated using thermogravimetric analysis (TGA, model TAQ500, Minneapolis, MN, USA). This experiment was conducted under dynamic conditions at a rate of 10 °C/min from 20 °C to 500 °C in an argon atmosphere (40 mL/min).

#### 2.4.2. Cell Proliferation

A water-soluble tetrazolium salt (WST-1) assay (Cell Proliferation Reagent, Roche, Basel, Switzerland) was conducted to evaluate cell proliferation at 24, 48, and 72 h, as well as at 7 days. Six biological replicates were utilized for each type of scaffold and study interval.

First, an initial density of 2 × 10^4^ cells/well was seeded onto the scaffolds and incubated in a humid atmosphere with 5% CO_2_ at 37 °C. After each study interval, the scaffolds were transferred into new wells containing 500 µL of culture media. The assay was conducted by adding 50 µL of WST-1 reagent and incubating under the same conditions for 4 h. The plates were gently vibrated for 1 min, and the supernatants were transferred to a new multi-well plate, which was measured using a microplate spectrophotometer (Multiskan SkyHigh, 2.00.35, Thermo Scientific, Waltham, MA, USA) at 440 nm (A_440_) and 650 nm (A_650_) as the reference wavelength following the manufacturer’s protocol. The cell proliferation was calculated as (A_440_–A_650_)—A_blank_, where A_blank_ represents the absorbance obtained from wells with scaffolds and culture media but without seeded cells. The average value of all biological replicates was recorded as the representative datum.

#### 2.4.3. Scanning Electron Microscopy (SEM)

A morphological analysis of the scaffold surfaces was conducted using SEM. An initial cell density of 2 × 10^4^ cells/well was seeded onto each scaffold in 48-multiwell plates and incubated in a humid atmosphere at 5% CO_2_ and 37 °C for 24 h and 7 days. Three biological replicates for each type of scaffold and study interval were utilized.

The cells were fixed with 2.5% glutaraldehyde plus 4% paraformaldehyde in PBS (pH 7.4). After each experimental timepoint, the culture medium was removed, and the scaffolds were completely submerged in the fixing solution for 2 h at room temperature. The fixing solution was then removed, and the scaffolds were kept in PBS overnight. The samples were then dehydrated using graded distilled water solutions (30—50—70—80—90—95% dH_2_O).

After dehydration, the specimens were dried using a critical point drying method following the manufacturer’s instructions (Leica EM CPD300, Leica Microsystems, Mannheim, Germany). The samples were subsequently sputtered with gold, and the scaffold topography was evaluated through SEM at a voltage of 7 kV (SEM-FEG Hitachi S-4800, Tokyo, Japan).

#### 2.4.4. Cell Viability—Confocal Laser Scanning Microscopy (CLSM)

Cell viability was assessed using CLSM (Leica Microsystems, Milton Keynes, United Kingdom) and the LIVE/DEAD© viability/cytotoxicity kit for mammalian cells (Invitrogen, Molecular Probes, 2005, Carlsbad, CA, USA). Three biological replicates were conducted for each type of scaffold and time point.

An initial density of 2 × 10^4^ cells/well was seeded onto the scaffolds using 48-well culture plates and incubated in a humid atmosphere at 5% CO_2_ and 37 °C for 24 h and 7 days. The scaffolds were washed with PBS to reduce esterase activity. Next, 20 µL of a 2 mM EthD-1 solution was added to 10 mL of PBS, yielding a final concentration of 4 µM EthD-1). Then, 5 µL of calcein AM was added to the solution and thoroughly mixed via pipetting. This produced a working solution containing 2 µM calcein AM and 4 µM EthD-1. A total of 150 µL of solution was added directly to each well, followed by a 30 min incubation at room temperature before visualization under CLSM following the manufacturer’s recommendations (Calcein AM: FITC filter, excitation 494 nm, emission 517 nm; EthD-1: RFP filter, excitation 528 nm, emission 617 nm).

A negative control, made up of scaffolds containing dead cells induced by incubation in 70% methanol for 15 min before CLSM analysis, was utilized to ensure the proper functioning of the resulting solution.

#### 2.4.5. Cytotoxicity

A direct cytotoxicity assay was performed, in which MG-63 cells were placed onto the scaffolds and cultured for 24 h, 48 h, 72 h, and 7 days. The assay used an initial cell density of 6 × 10^4^ cells/well, and a WST-1 assay (Cell Proliferation Reagent, Roche) was used to assess the cytotoxicity, following the protocol previously described (see cell proliferation assay).

#### 2.4.6. Gene Expression

To assess osteogenic-related gene expression, a reverse transcription-quantitative polymerase chain reaction (RT-qPCR) was performed. MG-63 cells were seeded onto the scaffolds at an initial density of 2–3 × 10^4^ cells/well and incubated for 24 h, 48 h, 72 h, and 7 days. Cells seeded directly onto the well were used as a control.

The total RNA was extracted using the RNeasy^®^ Mini Kit (Qiagen, Germantown, MD, USA) according to the standard protocol. The RNA quality and quantity were assessed through spectrophotometry (NanoDrop Thermo Scientific) to ensure a ratio of 260/230 ≥ 1.7. It was determined that the RNA obtained by pooling the genetic material from eight scaffolds would constitute one biological replicate. A total of 800 ng of total RNA was used for reverse transcription with the SuperScript™ IV VILO^™^ Master Mix kit (Invitrogen) and the ezDNase kit (Invitrogen) for gDNA elimination, following the manufacturer’s protocol. The qPCR analysis was conducted using KAPA SYBR^®^ FAST (KAPA BioSystems, Wilmington, MA, USA). Relative gene expression was analyzed using the 2^−ΔΔCt^ method and normalized to the reference genes TATA-box binding protein (TBP) and Glyceraldehyde 3-phosphate dehydrogenase (GAPDH) [26]. The primers used for PCR amplification are shown in Appendix A (Table A1).

#### 2.4.7. Protein Synthesis

The levels of matrix metalloproteinase 1 (MMP-1), receptor activator of nuclear factor kappa-B ligand (RANKL), macrophage colony-stimulating factor (M-CSF), interleukin (IL)-6, and IL-8 were measured using a multiplex immunoassay (Luminex200, Luminex Corporation, Austin, TX, USA).

MG-63 cells were seeded at an initial density of 2 × 10^4^ cells/well in 48 multi-well plates, which were incubated for 12 h to allow the cells to adhere to the scaffold surface. The scaffolds were then transferred to new plates and incubated for 24 h, 48 h, 72 h, and 7 days. Three biological replicates were conducted for each.

At each study interval, 500 µL of the supernatants were collected and stored at −20 °C until they were collectively analyzed. For quantifying the various proteins, three different kits were employed: MILLIPLEX^®^ Human Cytokine/Chemokine/Growth Factor Panel A (HCYTA-60K-03) for IL-6, IL-8, and M-CSF; MILLIPLEX^®^ MAP Human MMP Magnetic Bead Panel 2 (HMMP2MAG-55K-01) for MMP-1; and MILLIPLEX^®^ MAP Human RANKL Magnetic Bead—Single Plex (HRNKLMAG-51K-01) for RANKL.

Prior to the analysis, the samples were centrifuged to eliminate cell remnants, and the undiluted supernatants were analyzed according to the manufacturer’s protocol. The obtained data was assessed using xPonent software (version 4.2, Luminex Corporation, Austin, TX, USA).

### 2.5. Data Analysis

#### 2.5.1. Outcome Variables

Quantitative data were collected for the cell proliferation assay (measured in absorbance units (UAs)), cytotoxicity (measured in UAs), gene expression assay (measured as relative gene expression using the 2^−ΔΔCt^ method), and protein synthesis assay (measured in pg/mL).

#### 2.5.2. Statistical Analysis

Normality tests were carried out using the Shapiro–Wilk test and confirmed through distribution data of skewness, kurtosis, and boxplot diagrams. Data were presented as means and standard deviation (SD).

Comparisons within study intervals and between scaffolds and the control group were made using a one-way ANOVA test, followed by either Bonferroni’s posthoc test or Dunnet’s multiple comparisons test (based on the homogeneity of variances).

Statistical significance was established at *p* < 0.05. The analysis was conducted using IBM SPSS Statistics (version 29.0.1.1, IBM Corporation, New York, NY, USA).

## 3. Results

In all assays, a minimum sample size of three scaffolds for each infill density was tested. No cell culture contamination was observed during any of the assays.

### 3.1. Sterilization Method

HA-PLA50 scaffolds showed no bacterial or fungal growth in any of the 3 sterilization methods tested. However, in HA-PLA70 scaffolds, positive fungal growth was detected for the D0 cultures under aerobic conditions, but only in scaffolds sterilized with 70% ethanol.

**Figure 2:**
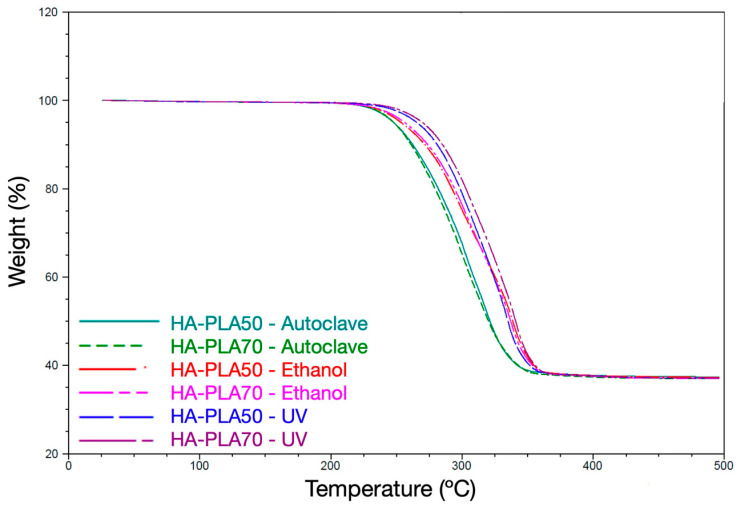
Thermogravimetry curves of HA-PLA50 and HA-PLA70 scaffolds after exposure to different sterilization methods. A higher rate of thermal degradation (represented as temperature-dependent weight loss) was detected for the scaffolds sterilized through autoclave, showing a faster weight loss with increasing temperatures.

The TGA test showed lower thermal degradation for scaffolds sterilized with UV radiation and 70% ethanol (Figure 2). However, scaffolds sterilized by autoclave showed a temperature degradation decrease of around 10–20 °C.

### 3.2. Cell Proliferation

**Figure 3:**
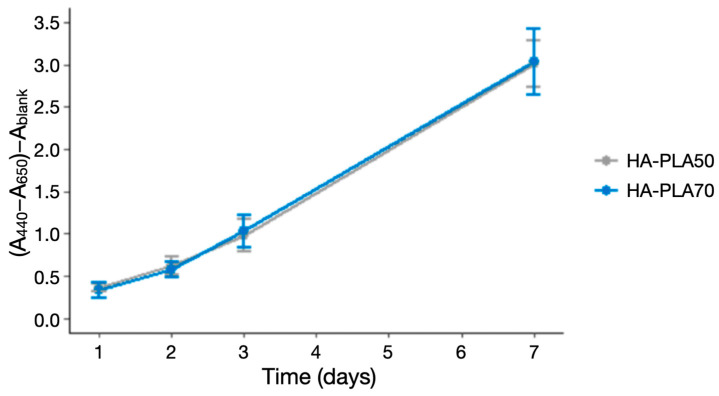
Cell proliferation of HA-PLA50 and HA-PLA70 scaffolds.

A similar cell proliferation rate was observed for both types of scaffolds, with a mean absorbance at 24 h of 0.33 AU (SD = 0.09) for HA-PLA70 and 0.36 AU (SD = 0.04) for HA-PLA50 (*p* > 0.05). At 7 days, the mean absorbance was 3.01 AU (SD = 0.28) for HA-PLA50 and 3.04 AU (SD = 0.39) for HA-PLA70 (*p* > 0.05) (Table 1) (Figure 3).

### 3.3. Scanning Electron Microscopy

**Figure 4:**
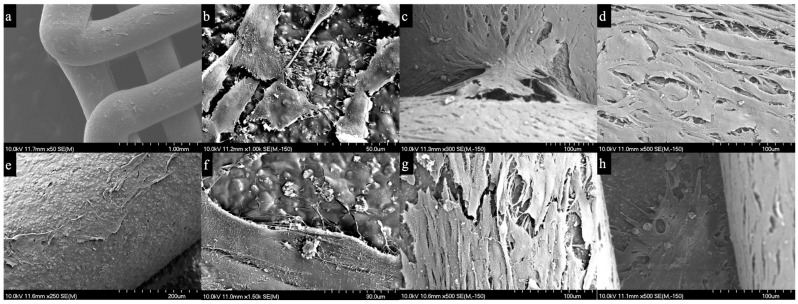
Scanning electron microscopy of HA-PLA50 (**a**–**d**) and HA-PLA70 (**e**–**h**) scaffolds seeded with MG-63 cells. (**a**) HA-PLA50 scaffolds at 24 h post-seeding at ×50 magnification. (**b**) HA-PLA50 scaffolds at 24 h post-seeding at ×1000 magnification. (**c**) HA-PLA50 scaffolds at 7 days post-seeding at ×300 magnification. (**d**) HA-PLA50 scaffolds at 7 days post-seeding at ×500 magnification. (**e**) HA-PLA70 scaffolds at 24 h post-seeding at ×250 magnification. (**f**) HA-PLA70 scaffolds at 24 h post-seeding at ×1500 magnification. (**g**,**h**) HA-PLA70 scaffolds at 7 days post-seeding at ×500 magnification. MG-63 cells exhibited a homogeneous distribution across the scaffold surface, characterized by a spindle-shaped morphology and cytoplasmic processes connecting the cells to one another and to the scaffold surface.

No differences in cell growth were observed between the scaffolds. The morphological features of the HA-PLA50 and HA-PLA70 scaffolds are shown in Figure 4. At 24 h, the scaffold surface exhibited a rough texture due to the HA particle topography, lacking micropores, and there was cell proliferation along it. The cells displayed a fibroblast-like morphology with cytoplasmic processes leading to interconnections between cells and connections to the scaffold. By day 7, the cell growth resulted in the formation of a dense cell layer over the scaffold surface.

### 3.4. Cell Viability–CLSM

**Figure 5:**
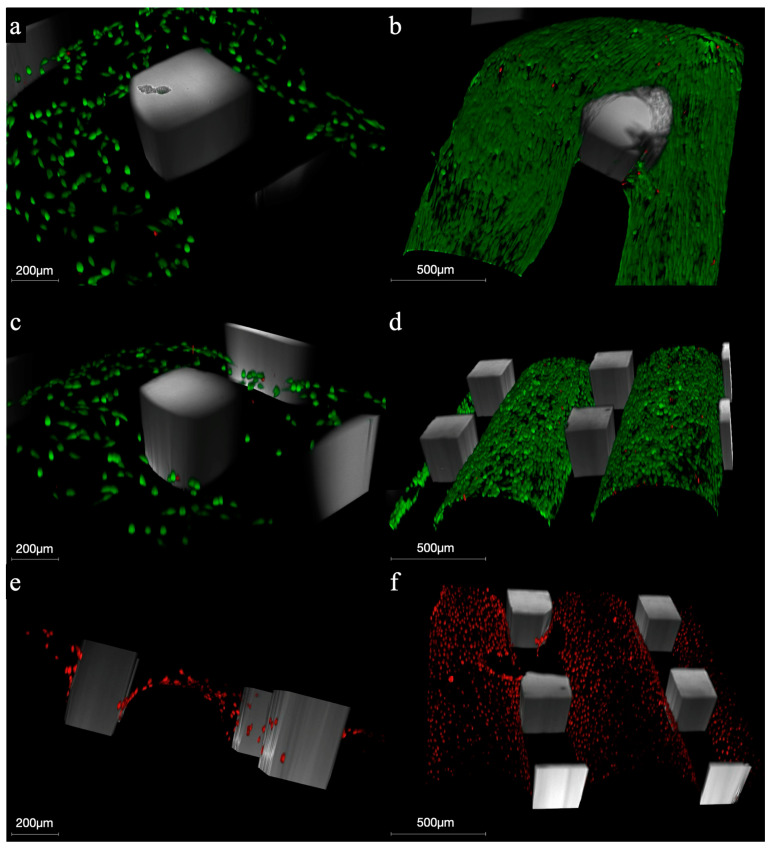
Confocal scanning microscopy of MG-63 cells stained with LIVE/DEAD dye, seeded onto HA-PLA50 and HA-PLA70 scaffolds after 24 h and 7 days of incubation. Both HA-PLA50 and HA-PLA70 scaffolds demonstrated high cell viability over the 7-day period. HA-PLA50 scaffolds: (**a**) 24 h; (**b**) 7 days. HA-PLA70 scaffolds: (**c**) 24 h; (**d**) 7 days. Control HA-PLA70 scaffolds: (**e**) 24 h; (**f**) 7 days. Live cells are shown in green and dead cells in red, with a magnification of 10×.

At 24 h, MG-63 cells were uniformly scattered along the surfaces of the HA-PLA50 and HA-PLA70 scaffolds, showing a low rate of dead cells. At 7 days, both scaffolds exhibited increased cell growth with a low death rate (Figure 5).

### 3.5. Cytotoxicity

In the direct cytotoxicity assay, no statistically significant changes in cytotoxicity were observed when comparing baseline and final time points for any of the scaffold’s porosities, nor when comparing between the two groups (*p* > 0.05) (Table 2).

### 3.6. Gene Expression

**Figure 6:**
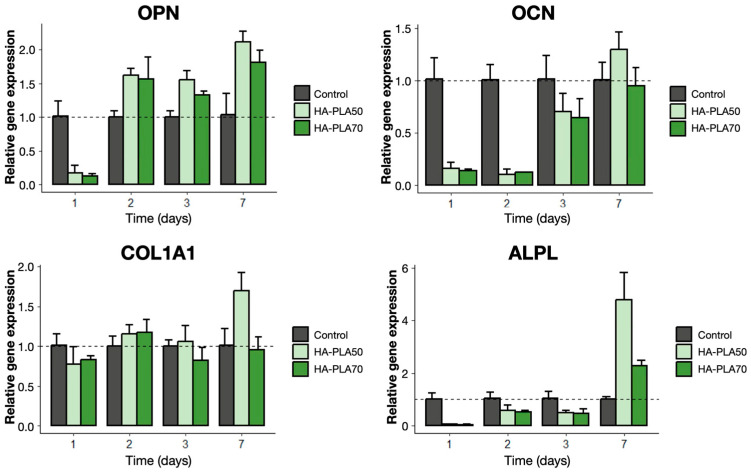
Relative gene expression of genes OPN, OCN, COL1A1, and ALPL, using GADPH and TBP as housekeeping genes, measured through RT-qPCR at 24 h, 48 h, 72 h, and 7 days. MG-63 cells growing on the well surface served as a positive control. OPN: osteopontin; OCN: osteocalcin; COL1A1: collagen type 1; ALPL: alkaline phosphatase.

Both types of scaffolds supported the growth of MG-63 cells, prompting the expression of OCN, OPN, ALPL, and COL1A1 at the mRNA level (Figure 6).

For HA-PLA50 scaffolds, COL1A1, OCN, and OPN exhibited a significant increase in gene expression when comparing baseline and final time points (*p* < 0.05). In the case of HA-PLA70 scaffolds, there was a significant increase in gene expression over time for ALPL and OPN (*p* < 0.05). When comparing both types of scaffolds, statistically significant differences at the final time point could only be observed for the COL1A1 gene, favoring the HA-PLA50 group (1.70 [SD = 0.22] vs. 0.96 [SD = 0.16], at the 7-day interval) (*p* < 0.05) (Table A2).

### 3.7. Protein Synthesis

**Figure 7:**
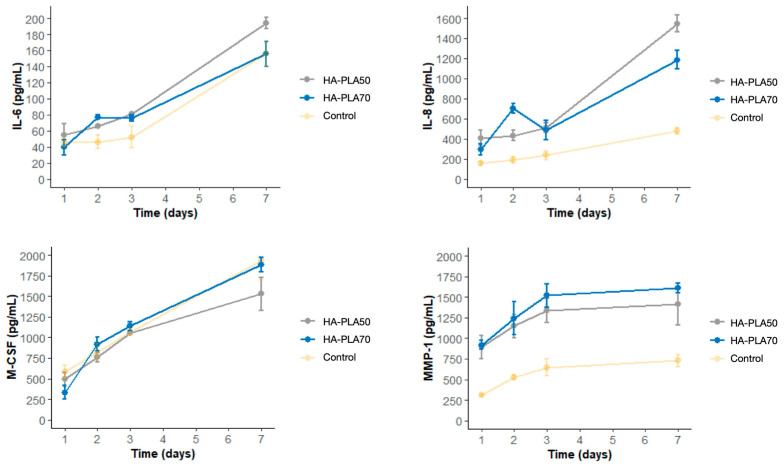
Protein synthesis in MG-63 cells for IL-6, IL-8, M-CSF, and MMP-1 at 24 h, 48 h, 72 h, and 7 days of incubation. This was measured using multiple immunoassays (Luminex^®^ 200) on supernatants. The concentrations of RANKL were also evaluated; however, the levels obtained at each study interval did not reach detectable limits. MG-63 cells growing on the well surface served as a positive control. IL-6: interleukin 6; IL-8: interleukin 8; M-CSF: macrophage colony-stimulating factor; MMP-1: metalloproteinase 1.

Out of five analytes, only MMP-1, IL-6, IL-8, and M-CSF produced detectable concentrations in the immunoassay analysis. RANKL concentrations remained below detection levels after the maximum incubation times (Figure 7).

All detectable analytes showed a significant increase (*p* < 0.05) in both types of scaffolds, except for MMP-1 in HA-PLA50 scaffolds, which revealed no statistically significant differences when comparing the baseline and final evaluations (Table A3).

When both scaffolds were compared, both IL-6 and IL-8 showed statistically significant differences, with higher concentrations for the HA-PLA50 scaffold (IL-6: 194.04 pg/mL [SD = 6.72] vs. 120.65 pg/mL [SD = 5.58] at the 7-day interval; IL-8: 1546.95 pg/mL [SD = 81.09] vs. 1187.54 pg/mL [SD = 90.89] at the 7-day interval) (*p* < 0.05). However, no differences between either type of scaffold or the control group were found for IL-6 (*p* > 0.05).

## 4. Discussion

This in vitro study evaluated the biological properties of two HA-PLA scaffolds with varying porosities, concentrating on their potential applications in tissue engineering for bone regeneration.

The evaluation of the most appropriate sterilization method identified ultraviolet radiation as the preferred technique for achieving successful sterilization while maintaining the integrity of the scaffolds. The use of MG-63 cells demonstrated successful cell proliferation on the surface of both scaffolds, confirming similar cell viability and proliferation on both types of scaffolds. Additionally, there were no differences in cytotoxicity between groups. Compared to HA-PLA70, the lower infill density (HA-PLA50) enhanced the expression of COL1A1. The protein concentrations of IL-8 at the final evaluation were also significantly higher in HA-PLA50 scaffolds compared to HA-PLA70 scaffolds. These results confirm adequate cell viability and proliferation of both tested HA-PLA scaffolds, although the one with lower infill density promoted a higher expression of osteogenic genes and IL-8, a significant chemokine and protein-binding cytokine.

Regarding the sterilization method, ethanol, UV radiation, and autoclave were selected for widespread adoption, especially for in vitro purposes since these scaffolds are designed for their use as point-of-care customized devices in craniofacial regeneration. The scaffolds were successfully sterilized without structural compromise using a 30 min exposure time per side of UV radiation. In fact, UV radiation is especially suited for biodegradable biomaterials like HA-PLA, as it minimizes risks of structural degradation or mechanical properties alterations associated with harsher sterilization techniques like autoclaving. However, there is still a lack of consensus on the optimal sterilization method for biodegradable scaffolds [27].

To analyze the biological performance of the scaffolds, we used MG-63 osteoblast-like cells to test cell viability and behavior when deposited on the scaffold surfaces. Both SEM and CSLM analyses confirmed adequate cell growth and adhesion over a 7-day incubation period, indicating that MG-63 cells adhered to both scaffold surfaces and successfully proliferated. Cells growing on both types of scaffolds exhibited a similar morphology, with cytoplasmic processes or filopodia interconnecting the cells and the material surface. This observation aligns with studies reporting similar experiments where MG-63 cells reached complete confluence over PLA scaffolds after 14 days [28,29,30,31]. Consistent with our approach, multiple investigations have evaluated the effect of scaffold structure and surface on cell proliferation and differentiation. For instance, Pamula et al. observed that for poly(L-lactide-co-glycolide), scaffolds with 83% porosity showed faster initial cell colonization during the first week of incubation in scaffolds featuring larger pore diameters (400 to 600 µm); however, this difference disappeared at the 2-week assessment [32]. Our results similarly highlight the importance of structural parameters, while extending these observations to HA-PLA composites with controlled infill densities.

The cytotoxicity assessment revealed no statistically significant differences between HAP-PLA50 and HA-PLA70 scaffolds at any timepoint nor across incubation periods within each scaffold. Similar results were reported by Gregor et al. using MG-63 cells, where no differences in cytotoxicity were observed among PLA scaffolds with varying porosities during a 5-day incubation period [30]. Therefore, both HA-PLA50 and HA-PLA70 scaffolds can be considered non-toxic to MG-63 cells.

The effects of infill density, porosity, and scaffold design on scaffold performance and cell behavior have been thoroughly examined in biomaterials research. This study observed variations in osteogenic gene expression and protein synthesis associated with lower infill densities (HA-PLA50 scaffolds). Chocholata et al. (2019) noted that during scaffold design, the pore size should be sufficiently large to allow for cell migration while remaining small enough to promote cell binding to the scaffold surface [11]. Huang et al. reported that PLA-nanoHA scaffolds with similar porosities (80%) showed enhanced cell adhesion and proliferation [13]. In this study, the expression of osteogenesis-related genes by MG-63 cells cultured on both types of scaffolds indicated sufficient biocompatibility and osteogenic capacity. HA-PLA50 scaffolds demonstrated a higher relative gene expression of COL1A1 compared to the higher porosity scaffolds. These findings are consistent with Gregor et al.’s results, which noted an increased collagen type 1 formation after 7 days in PLA scaffolds [30]. Therefore, these results suggest that infill density may influence cell adhesion, proliferation, and differentiation towards an osteogenic lineage.

In addition to gene expression, the study also examined protein synthesis, focusing on the secretion of different osteogenic markers. The protein concentrations of IL-8 were significantly higher in the HA-PLA50 scaffolds compared to the HA-PLA70 scaffolds. Interestingly, while elevated IL-8 could be associated with an increased immune response, this cytokine’s high protein-binding activity could also reflect the scaffolds’ potential to promote the recruitment of cells involved in the wound healing process and tissue regeneration, such as endothelial and immune-competent cells. Overall, the lower infill density of HA-PLA50 likely facilitated nutrient diffusion and cell–matrix interactions, explaining its superior protein/gene expression profile compared to denser HA-PLA70.

We recognize the limitations of this study, particularly the reduced sample size for each assay, dictated by limited biomaterial availability. The maximum incubation time for the experiments was set at 7 days to reduce the risk of microbial contamination; however, this may limit the insights gained from a longer degradation period. While SEM and CLSM demonstrated adequate cell–scaffold integration qualitatively, a quantitative morphometric analysis was not performed. Similarly, the assessment of osteogenic potential was conducted using molecular markers rather than histological staining (ALP/ARS), which could have provided additional data on mineralization. Additionally, the in vitro nature of this investigation does not allow for proper translation to more complex living models, emphasizing the need for in vivo and clinical studies to confirm these findings. Several 3D-printed devices have already been tested in rabbits [29], rat models [13,33], and ovine models [34], and recent case reports have shown successful outcomes in human regeneration of bone lesions in the jaw [35]. Nevertheless, despite these encouraging results, the evidence remains limited, and regulatory considerations continue to restrict their use in clinical settings [7].

However, the results of this investigation are relevant for future research using in vivo models, as 3D-printed synthetic composite bone replacement grafts provide highly suitable biological and mechanical properties for clinical applications. More specifically, HA-PLA50 and HA-PLA70 scaffolds have demonstrated biocompatibility and bioactivity when studied under strictly controlled experimental conditions. While these in vitro results offer initial evidence of their potential for tissue engineering applications, future research should evaluate the performance of these materials in vivo, where the complex interactions between the scaffold, host tissue, and immune response can be assessed more comprehensively.

## 5. Conclusions

This study demonstrates that both HA-PLA50 and HA-PLA70 scaffolds exhibit adequate biocompatibility, non-toxicity, and osteogenic potential in vitro, supporting their suitability for applications in bone regeneration.

Our key findings are as follows: (1) ultraviolet radiation is the optimal sterilization method, preserving scaffold integrity while ensuring cell viability; (2) both scaffolds supported significant MG-63 cell proliferation over 7 days, with no differences in cytotoxicity observed; (3) HA-PLA50 scaffolds outperformed HA-PLA70 in COL1A1 gene expression and synthesis of IL-6 and IL-8.

The HA-PLA50 and HA-PLA70 scaffolds have demonstrated biocompatibility and bioactivity when studied under strictly controlled experimental conditions. While these in vitro results offer initial evidence of their potential for tissue engineering applications, future research should evaluate the performance of these materials in vivo, where the complex interactions between the scaffold, host tissue, and immune response can be assessed more comprehensively.

## Figures and Tables

**Table 1:** Cell proliferation of HA-PLA50 and HA-PLA70 scaffolds measured by spectrophotometry using a WST-1 tetrazolium assay, measured at 24 h, 48 h, 72 h, and 7 days. No statistical differences between scaffolds were found for any of the study intervals. One-way ANOVA test and Dunnet’s multiple comparisons test.

Infill	N	Absorbance (Mean (SD))				*p* Value (Between Time Points)		
		24 h	48 h	72 h	1 W	24 h vs. 48 h	24 h vs. 72 h	24 h vs. 1 W
HA-PLA50	6	0.36 (0.04)	0.62 (0.10)	0.96 (0.19)	3.01 (0.28)	0.014	0.006	<0.001
HA-PLA70	6	0.33 (0.09)	0.57 (0.09)	1.01 (0.19)	3.04 (0.39)	0.020	0.001	<0.001
*p* value (between scaffolds)		1.000	1.000	1.000	1.000			

**Table 2:** Direct cytotoxicity assay using a WST-1 tetrazolium assay, measured through spectrophotometry at 24 h, 48 h, 72 h, and 7 days. One-way ANOVA test and Dunnet’s multiple comparisons test.

Infill	N	Absorbance (Mean (SD))				*p* Value (Between Time Points)		
		24 h	48 h	72 h	1 W	24 h vs. 48 h	24 h vs. 72 h	24 h vs. 1 W
HA-PLA50	6	1.94 (0.28)	1.94 (0.20)	2.41 (0.51)	2.21 (0.11)	1.000	0.716	0.666
HA-PLA70	6	2.56 (0.19)	1.66 (0.50)	2.40 (0.37)	2.12 (0.17)	0.080	1.000	0.177
*p* value (between scaffolds)		0.071	0.974	1.000	0.999			

## Data Availability

The original contributions presented in the study are included in the article, further inquiries can be directed to the corresponding authors.

## Abbreviations

The following abbreviations are used in this manuscript:

CLSM	Confocal scanning laser microscopy
COL1A1	Collagen type 1
GAPDH	Glyceraldehyde 3-phosphate dehydrogenase
HA	Hydroxyapatite
IL	Interleukin
M-CSF	Macrophage colony-stimulating factor
MMP	Matrix metalloproteinase
PLA	Polylactic acid
RANKL	Receptor activator of nuclear factor kappa-B ligand
RT-qPCR	Reverse transcription-quantitative polymerase chain reaction
SEM	Scanning electron microscopy
TBP	TATA-box binding protein
TGA	Thermogravimetry
UV	Ultraviolet
WST	Water-soluble tetrazolium

## Appendix A

**Table A1:** Primer sequences used to amplify each target and reference gene by RT-qPCR.

Gene	Primer	Sequence
TBP	Forward	5′-TGTATCCACAGTGAATCTTGGTTG-3′
	Reverse	5′-GGTTCGTGGCTCTCTTATCCTC-3′
GAPDH	Forward	5′-GTCTCCTCTGACTTCAACAGCG-3′
	Reverse	5′-ACCACCCTGTTGCTGTAGCCAA-3′
OPN	Forward	5′-CGAGGTGATATAGTGTGGTTTATGG-3′
	Reverse	5′-GCACCATTCAACTCCTCGCTTTC-3′
OCN	Forward	5′-CGCTACCTGTATCAATGGCTGG-3′
	Reverse	5′-CTCCTGAAAGCCGATGTGGTCA-3′
COL1A1	Forward	5′-GATTCCCTGGACCTAAAGGTGC-3′
	Reverse	5′-AGCCTCTCCATCTTTGCCAGCA-3′
ALPL	Forward	5′-GCTGTAAGGACATCGCCTACCA-3′
	Reverse	5′-CCTGGCTTTCTCGTCACTCTCA-3′

**Table A2:** Relative gene expression of OPN, OCN, COL1A1, and ALPL, using GADPH and TBP as housekeeping genes, measured through RT-qPCR at 24 h, 48 h, 72 h, and 7 days. One-way ANOVA and Bonferroni tests (for COL1A1 and OPN) and Dunnett’s multiple comparisons test (for ALPL and OCN) were conducted.

Gene	Infill	N		Relative Gene Expression (Mean (SD))				*p* Value (Between Time Points)		
				24 h	48 h	72 h	1W	24 h vs. 48 h	24 h vs. 72 h	24 h vs. 1 W
ALPL	HA-PLA50	3		0.05 (0.01)	0.57 (0.21)	0.49 (0.08)	4.77 (1.04)	0.317	0.069	0.107
	HA-PLA70	3		0.04 (0.01)	0.50 (0.06)	0.47 (0.17)	2.28 (0.19)	0.033	0.294	0.017
	CONTROL	3		1.01 (0.22)	1.02 (0.25)	1.02 (0.26)	1.00 (0.08)	1.000	1.000	1.000
	*p* value (between scaffolds)	HA-PLA50 vs. HA-PLA70		0.034	1.000	1.000	0.326			
		HA-PLA50 vs. Control		0.324	0.681	0.414	0.161			
		HA-PLA70 vs. Control		0.320	0.409	0.438	0.032			
COL1A1	HA-PLA50	3		0.77 (0.21)	1.16 (0.11)	1.06 (0.21)	1.70 (0.22)	0.528	1.000	<0.001
	HA-PLA70	3		0.83 (0.05)	1.17 (0.16)	0.82 (0.16)	0.96 (0.16)	1.000	1.000	1.000
	CONTROL	3		1.01 (0.15)	1.00 (0.12)	1.00 (0.07)	1.02 (0.21)	1.000	1.000	1.000
	*p* value (between scaffolds)	HA-PLA50 vs. HA-PLA70		1.000	1.000	1.000	<0.001			
		HA-PLA50 vs. Control		1.000	1.000	1.000	0.002			
		HA-PLA70 vs. Control		1.000	1.000	1.000	1.000			
OCN	HA-PLA50	3		0.15 (0.06)	0.10 (0.05)	0.70 (0.17)	1.29 (0.17)	0.998	0.185	0.030
	HA-PLA70	3		0.13 (0.02)	0.12 (0.00)	0.64 (0.18)	0.95 (0.17)	0.985	0.255	0.098
	CONTROL	3		1.01 (0.20)	1.00 (0.14)	1.01 (0.22)	1.00 (0.16)	1.000	1.000	1.000
	*p* value (between scaffolds)	HA-PLA50 vs. HA-PLA70		1.000	1.000	1.000	0.632			
		HA-PLA50 vs. Control		0.100	0.040	0.843	0.841			
		HA-PLA70 vs. Control		0.113	0.062	0.732	1.000			
OPN	HA-PLA50	3		0.16 (0.11)	1.62 (0,09)	1.55 (0.14)	2.11 (0.16)	<0.001	<0.001	<0.001
	HA-PLA70	3		0.12 (0.03)	1.57 (0.32)	1.33 (0.05)	1.81 (0.19)	<0.001	<0.001	<0.001
	CONTROL	3		1.01 (0.22)	1.00 (0.09)	1.00 (0.09)	1.03 (0.32)	1.000	1.000	1.000
	*p* value (between scaffolds)	HA-PLA50 vs. HA-PLA70		1.000	1.000	1.000	1.000			
		HA-PLA50 vs. Control		<0.001	0.024	0.182	<0.001			
		HA-PLA70 vs. Control		<0.001	0.057	1.000	0.002			

**Table A3:** Protein synthesis of MG-63 cells for IL-6, IL-8, M-CSF, and MMP-1 was measured at 24 h, 48 h, 72 h, and 7 days of incubation using multiple immunoassays (Luminex^®^). For IL-8, a one-way ANOVA test and Bonferroni test were applied, while Dunnett’s multiple comparisons test was utilized for IL-6 and M-CSF MMP-1.

Protein Synthesis	Infill	N	Concentration pg/mL (Mean (SD))				*p* Value (Between Time Points)		
			24 h	48 h	72 h	1 W	24 h vs. 48 h	24 h vs. 72 h	24 h vs. 1 W
IL-6	HA-PLA50	3	55.25 (13.32)	66.09 (1.09)	80.68 (1.14)	194.04 (6.72)	0.954	0.461	0.007
	HA-PLA70	3	39.33 (9.37)	77.07 (2.47)	75.66 (4.28)	120.65 (5.58)	0.117	0.107	0.009
	CONTROL	3	45.40 (6.05)	46.18 (8.13)	52.22 (13.42)	155.57 (15.62)	1.000	1.000	0.027
	*p* value (between scaffolds)	HA-PLA50 vs. HA-PLA70	0.916	0.077	0.813	0.003			
		HA-PLA50 vs. Control	0.992	0.314	0.395	0.300			
		HA-PLA70 vs. Control	1.000	0.128	0.533	0.360			
IL-8	HA-PLA50	3	408.04 (75.85)	434.42 (48.83)	510.72 (48.75)	1546.95 (81.09)	1.000	1.000	<0.001
	HA-PLA70	3	293.65 (56.01)	701.98 (50.19)	484.16 (95.73)	1187.54 (90.89)	<0.001	0.051	<0.001
	CONTROL	3	155.10 (20.08)	188.73 (30.11)	234.43 (41.37)	479.21 (29.63)	1.000	1.000	<0.001
	*p* value (between scaffolds)	HA-PLA50 vs. HA-PLA70	1.000	<0.001	1.000	<0.001			
		HA-PLA50 vs. Control	0.002	0.003	<0.001	<0.001			
		HA-PLA70 vs. Control	0.655	<0.001	0.002	<0.001			
M-CSF	HA-PLA50	3	492.79 (84.50)	755.61 (59.26)	1055.86 (6.84)	3054.39 (396.82)	0.183	0.050	0.046
	HA-PLA70	3	333.43 (80.04)	918.58 (84.30)	1137.25 (55.17)	3756.68 (175.99)	0.016	0.004	0.001
	CONTROL	3	582.21 (74.77)	821.08 (100.85)	1065.47 (31.64)	3870.45 (477.06)	0.371	0.031	0.043
	*p* value (between scaffolds)	HA-PLA50 vs. HA-PLA70	0.673	0.539	0.640	0.542			
		HA-PLA50 vs. Control	0.981	0.999	1.000	0.710			
		HA-PLA70 vs. Control	0.229	0.989	0.827	1.000			
MMP-1	HA-PLA50	3	893.41 (140.99)	1146.76 (143.42)	1333.12 (145.00)	2823.84 (513.31)	0.749	0.257	0.133
	HA-PLA70	3	919.62 (56.51)	1242.56 (200.89)	1519.94 (140.59)	3212.64 (114.59)	0.592	0.089	0.001
	CONTROL	3	307.15 (17.50)	525.09 (30.18)	646.04 (101.22)	1451.68 (138.29)	0.016	0.178	0.031
	*p* value (between scaffolds)	HA-PLA50 vs. HA-PLA70	1.000	1.000	0.944	0.976			
		HA-PLA50 vs. Control	0.121	0.105	0.052	0.262			
		HA-PLA70 vs. Control	0.012	0.159	0.021	0.002			
